# Resistome Analysis of Global Livestock and Soil Microbiomes

**DOI:** 10.3389/fmicb.2022.897905

**Published:** 2022-07-07

**Authors:** Katie Lawther, Fernanda Godoy Santos, Linda Boniface Oyama, Francesco Rubino, Steven Morrison, Chris J. Creevey, John W. McGrath, Sharon Ann Huws

**Affiliations:** ^1^School of Biological Sciences, Institute for Global Food Security, Queen’s University Belfast, Belfast, United Kingdom; ^2^Agri-Food and Biosciences Institute, Belfast, United Kingdom

**Keywords:** ruminant, poultry, swine, soil, metagenome, resistome, antimicrobial resistance gene, antimicrobial resistance

## Abstract

Antimicrobial resistance (AMR) is a serious threat to public health globally; it is estimated that AMR bacteria caused 1.27 million deaths in 2019, and this is set to rise to 10 million deaths annually. Agricultural and soil environments act as antimicrobial resistance gene (ARG) reservoirs, operating as a link between different ecosystems and enabling the mixing and dissemination of resistance genes. Due to the close interactions between humans and agricultural environments, these AMR gene reservoirs are a major risk to both human and animal health. In this study, we aimed to identify the resistance gene reservoirs present in four microbiomes: poultry, ruminant, swine gastrointestinal (GI) tracts coupled with those from soil. This large study brings together every poultry, swine, ruminant, and soil shotgun metagenomic sequence available on the NCBI sequence read archive for the first time. We use the ResFinder database to identify acquired antimicrobial resistance genes in over 5,800 metagenomes. ARGs were diverse and widespread within the metagenomes, with 235, 101, 167, and 182 different resistance genes identified in the poultry, ruminant, swine, and soil microbiomes, respectively. The tetracycline resistance genes were the most widespread in the livestock GI microbiomes, including *tet*(W)_1, *tet*(Q)_1, *tet*(O)_1, and *tet*(44)_1. The *tet*(W)_1 resistance gene was found in 99% of livestock GI tract microbiomes, while *tet*(Q)_1 was identified in 93%, *tet*(O)_1 in 82%, and finally *tet*(44)_1 in 69%. Metatranscriptomic analysis confirmed these genes were “real” and expressed in one or more of the livestock GI tract microbiomes, with *tet*(40)_1 and *tet*(O)_1 expressed in all three livestock microbiomes. In soil, the most abundant ARG was the oleandomycin resistance gene, *ole*(B)_1. A total of 55 resistance genes were shared by the four microbiomes, with 11 ARGs actively expressed in two or more microbiomes. By using all available metagenomes we were able to mine a large number of samples and describe resistomes in 37 countries. This study provides a global insight into the diverse and abundant antimicrobial resistance gene reservoirs present in both livestock and soil microbiomes.

## Introduction

It has been 20 years since, the WHO published their first “Global Strategy for Containment of Antimicrobial Resistance (AMR),” AMR remains a serious threat to both animal and human health as well as the world’s economy (WHO, 2001, 2021). The Centre for Disease Control and Prevention (CDC) estimates an annual cost of $55 billion dollars associated with AMR within the United States, including additional healthcare costs of $20 billion (CDC, 2013). Over 2.8 million antibiotic-resistance infections occur annually in the United States, and treatments can be lengthy and expensive. For example, each case of extensively drug-resistant tuberculosis costs $526,000 to treat, and extended-spectrum beta-lactamase (ESBL) producing *Enterobacteriaceae* cost $1.2 billion in healthcare costs per year (CDC, 2019). Within the United Kingdom, there were an estimated 65,162 resistant infections in 2019, an increase of over 3,000 compared to the previous year [English Surveillance Programme for Antimicrobial Utilisation and Resistance (ESPAUR) Report, 2020], with 21% of all key pathogen bloodstream infections reported in England showing resistance to at least one antibiotic [English Surveillance Programme for Antimicrobial Utilisation and Resistance (ESPAUR) Report, 2020]. By 2050, it is estimated that AMR will have cost 100 trillion USD from the world’s economy (O’Neill, 2016). Globally, 1.27 million deaths were caused by bacterial AMR in 2019 (Antimicrobial Resistance Collaborators et al., 2022) with up to 10 million deaths predicted to occur annually by 2050 (O’Neill, 2016).

Surveillance using a “One Health” approach, covering the use of antibiotics and resistance rates within humans, animals, and the environment in which they coexist, is essential for effectively assessing the spread of AMR (WHO, 2001, 2020; Zinsstag et al., 2011). This approach considers the close interactions between humans, animals, and the environment, with the World Organisation for Animal Health, the Food and Agriculture Organization of the United Nations, and the WHO recognizing AMR as a critical issue requiring such a One Health surveillance strategy (Zinsstag et al., 2011). As an example, humans can be exposed to AMR pathogens through the food chain, including through consumption of contaminated meat leading to food borne illnesses (Zhao et al., 2008). Additionally, a high exchange frequency of mobile antimicrobial resistance genes (ARGs) can occur between bacteria that infect animals and humans (Hu et al., 2016). Human–animal-shared mobile ARGs have been identified in human, chicken, pig, and cattle guts, conferring resistance to six major antibiotic classes: tetrayclcines, aminoglycosides, macrolide-lincosamide-streptogramin B (MLSB), chloramphenicols, β-lactams, and sulphonamides. These ARGs that transfer between human and animal guts are also harbored by human pathogens (Hu et al., 2016). Additionally, there is a shared resistome between soil bacteria and human pathogens, and it has been suggested that the soil microbiota act as a reservoir of resistance genes (Forsberg et al., 2012). Such exchange can happen *via* horizontal gene transfer; these mechanisms allow bacteria to exchange genes including ARGs, making both inter- and intraspecies exchange possible. This allows for the development of mobile genetic elements such as plasmids which include multiple ARGs, leading to multidrug resistant or extensively drug-resistant bacteria; these plasmids can transfer to different bacteria in various environments (Sun et al., 2019).

Antibiotics are commonly administered to livestock, particularly in countries where prophylactic use is allowed. For example over 17 million kg of antimicrobials sold in the United States in 2019 was for use in food-producing animals, with over one-third of this amount being antimicrobials important to human health (FDA, 2020). The BRICS countries (Brazil, Russia, India, China, and South Africa) are estimated to double their antimicrobial use in animals by 2030, and the use of antimicrobials applied to chickens is set to triple in India by the same year (Van Boeckel et al., 2015). Intensive farming conditions, including high animal densities in indoor production systems and poor biosecurity (as typically seen in monogastric farming), can lead to livestock being more vulnerable to disease thus leading to even higher antimicrobial usage (FAO, 2016). Antimicrobials can also be widely abused due to their low cost and over the counter availability in some countries (FAO, 2016). Such practices can lead to excess antibiotic use, which places a selection pressure on the microbiota within the animal gut flora, leading to an alteration of the diversity of the host microbiota and the promotion of exchange of genetic material (FAO, 2016).

Antimicrobial resistance within livestock farming will also impact AMR in the environment as well as human and animal health. A significant quantity of those antimicrobials applied to animals are thought to be largely unmetabolized, with up to 90% of antibiotics used in food animals being excreted (Marshall and Levy, 2011). Tetracycline applied to pigs has been shown to be rapidly excreted, with up to 72% of the active ingredient recovered in the animals’ feces and urine (Winckler and Grafe, 2001). While soil microbiota do naturally harbor ARGs, known as the intrinsic resistome (Zhu et al., 2019), feces and urine of grazing animals and the application of animal manure as fertilizer can lead to the dissemination of resistance in soil bacteria (Ghosh and LaPara, 2007; Iwu et al., 2020). For example, Zhu et al. (2013) have shown that manure sourced from Chinese pig farms was enriched by up to 28,000 fold in ARGs with a concomitant 90,000-fold increase in transposons, when compared to antibiotic free manure and soil. Soil can also be exposed to antimicrobials, ARGs and resistant organisms through the application of sewage sludge for crop production and wastewater from industrial, agricultural, pharmaceutical, and municipal treatment plants for irrigation (Fouz et al., 2020). These exposures to antimicrobials can lead to changes in the diversity of the soil resistome; it has been suggested that microorganisms that have acquired resistance now dominate over the intrinsic resistant organisms (Zhu et al., 2019). Resistance genes identical in sequence to those found in a range of human pathogens have also been identified in environmental soil bacteria, including multidrug resistance cassettes, providing resistance against five different antibiotic classes (Forsberg et al., 2012).

Given the intrinsic link between animal manures, soil as a reservoir of ARGs (Forsberg et al., 2012), and the associated potential impact on human health, we now present a global surveillance study of acquired ARGs in the gastrointestinal tracts of ruminants, swine, and poultry and the soil environment. Additionally, we confirm that these ARGs were functional and expressed within metatranscriptomic datasets of the microbiomes from these environments. This study provides a global outlook of antimicrobial resistance across different microbiomes in livestock farming landscapes, and soil environments.

## Materials and Methods

### Selection of Metagenomic Data for Identification of ARGs

The National Center for Biotechnology Information (NCBI) Sequence Read Archive (SRA) (Leinonen et al., 2010) was searched for metagenomic sequencing datasets for the following microbiomes: ruminant (species *Bos Taurus*), poultry, swine, and soil. All available metagenomic data on the NCBI, as of July 31, 2019, for each microbiome were considered for this study. The search terms used are outlined in Table 1, with individual searches completed for each search term.

**Table 1:** NCBI SRA search terms.

Microbiome	Search term 1	Search term 2	Search term 3	Search term 4
Ruminant	Rumen gut	Rumen feces^*^	Bovine feces^*^	Bovine gut
Poultry	Poultry feces^*^	Poultry gut	Chicken gut	Chicken feces^*^
Swine	Swine gut	Swine feces^*^	Pig feces^*^	Pig gut
Soil	Soil			

Each search term was followed by the word metagenome.

* Feces/fecal/feces/fecal.

Bioprojects matching at least one of these search terms were filtered to only include whole shotgun metagenomic sequence data and sequencing performed on Illumina sequencing platforms to ensure consistency between samples, both single and paired end reads were included. Bioprojects were only included if they were of known geographical location, i.e., the country of sample origin. Bioprojects were excluded if they were genomic sequences from pure culture and 16S rDNA sequencing data, involved bench top or post sampling enrichment or involved animals participating in an antibiotic trial (as the purpose of this study was to investigate baseline resistance). The study design and abstract for each Bioproject on the NCBI SRA were checked and, where available, the associated papers were read. All biosamples, within the bioprojects that met these criteria, were included in the final dataset for analysis (Table 2).

**Table 2:** Sequencing datasets selected for analysis.

Microbiome	Total number of sequencing datasets	Total size of sequencing data (Gb)
Ruminant	592	4,794.3
Poultry	306	1,092.8
Swine	784	1,772.4
Soil	4,161	19,513.2

The total number of sequencing datasets and sizes of data used in this study collected from the NCBI SRA database.

### Identification of Antimicrobial Resistance Genes

All bioinformatic analyses described below were performed on the QUB High Performance Cluster system, Kelvin. All metagenomic sequences meeting selection criteria, as described above, were downloaded from the NCBI databases and local blast searches performed. Each sequence run was downloaded in compressed sra format (.sra) using the *prefetch* tool. The files were converted from sra to fasta format using the SRA-Toolkit version 2.9.6 (Leinonen et al., 2010). An AMR database file was created by downloading ARGs (excluding any ARGs due to point mutations) from the ResFinder database resulting in a fasta file containing 2,470 acquired ARG sequences (downloaded on July 31, 2019) (Zankari et al., 2012). To overcome the potential challenge that nucleotide redundancy in ARGs (with the same function) may lead to their separation using Resfinder, clustering was performed via CD-HIT-EST analysis using Galaxy version 1.2 (Fu et al., 2012; Afgan et al., 2018). This clustering avoided multiple ARGs of similar sequence being overrepresented by matching to the same stretch of metagenomic sequence clustering resulted in 302 ARGs. The clustering cut offs were 90% similarity and sequence length difference cutoff of 99%, i.e., the shorter sequences need to be at least 99% length of the representative of the cluster.

Using NCBI BLAST version 2.2.30, nucleotides searches were performed to identify the clustered ResFinder ARGs within the metagenomic sequences. Default BLASTn parameters and the output format six were used, outputting a tab-separated table with no preset column headers. A specific text file output was requested including the query accession, sequence accession, e-value, query start, query end, sequence start, sequence end, bitscore, percentage of identical matches, and alignment length (Madden, 2008).

**Figure 1:**
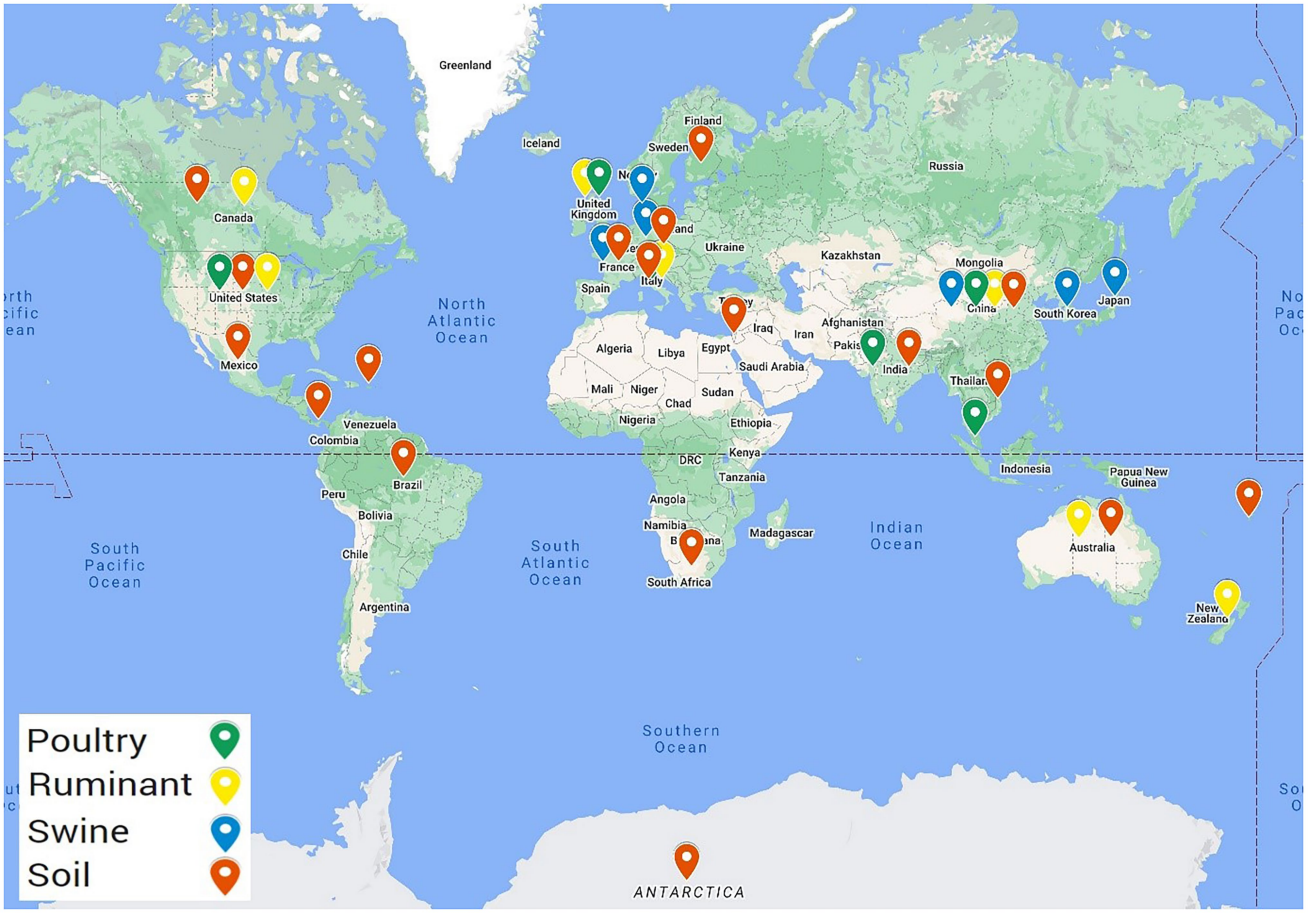
Geographical locations of the selected metagenomic samples containing ARGs. Metagenomes selected from NCBI SRA including 509 ruminant metagenomes from eight different countries, 277 poultry metagenomes from six countries, 710 swine metagenomes sourced from seven countries, and soil metagenomes from 23 countries.

Summary files were then created from the BLAST result files to filter the BLAST hits such that only those with a percentage identity ≥75%, and a bitscore of ≥60 were included. This was completed using a shell script for example, cat soil1.txt | awk ‘{if ($8 >= 60 && $9 >= 75) print $0}’ | sort -k2,2 | cut -f2 | uniq -c | sort -rn>summarysoil1.txt. The output included the incidence of the AMR gene in that sequence that met the criteria. Any metagenome fasta file (biosample) that had no AMR genes identified after applying the cut off filters were removed from this point and did not proceed for downstream analysis, this included 23 soil, five ruminant, two poultry, and two swine biosamples. Results were further filtered to remove low frequency hits, with ARG hits included if they appeared >25 times within the biosample. This filter was selected to balance the retention of data with the removal of background noise and low frequency hits (Supplementary Figure 1). Essentially, any gene that appeared >25 times within a sample was considered as present and denoted as 1, any gene present ≤25 times was considered absent, 0. All countries that contained AMR genes following application of these cutoffs are highlighted in Figure 1. Datasets from five countries including Argentina, Colombia, Madagascar, Papua New Guinea, and Sri Lanka did not contain ARGs meeting these cutoffs and were consequently excluded from further analysis.

### Metatranscriptomics: Gene Expression Analysis

In order to evaluate the potential functionality and active expression of identified ARGs, the ARG sequences were aligned to metatranscriptome datasets. Metatranscriptome datasets were selected for each of the four microbiomes: ruminant, poultry, swine, and soil. The metatranscriptomic studies for both gut microbiomes of swine and poultry were selected from the FAANG database,^1^ and the largest available metatranscriptomic datasets from published studies and free from antibiotic treatment were selected. These included the poultry metranscriptomic dataset: PRJEB23255, Germany (Reyer et al., 2018) and the swine datasets: PRJNA529662, PRJNA529214, United States of America (Keel et al., 2020). Similarly, the largest antibiotic free metatranscriptomic soil dataset was selected from the ENA database,^2^ PRJNA366008, United States of America (unpublished). These metatranscriptomic datasets were downloaded using *wget* version 1.14. Finally, to represent the ruminant category, the metatranscriptome data from the study by Shi et al. (2014) (PRJNA202380, New Zealand) were investigated.

Analysis was performed using Bowtie2 (version 2.3.5.1) and the methods described in Sabino et al. (2019) and Langmead and Salzberg (2012). In brief, the DNA sequences of ARGs contained on the ResFinder database were indexed using the Bowtie2-build tool. The downloaded metatranscriptomic datasets were then aligned to these indexes, default end-to-end alignment cut offs were used, and minimum score threshold was −0.6 + −0.6 * L, where L is the read length. The presence and absence of alignment to an ARG were then summarized from the SAM files produced from bowtie2.

### Statistics and Graphical Representation

**Figure 2:**
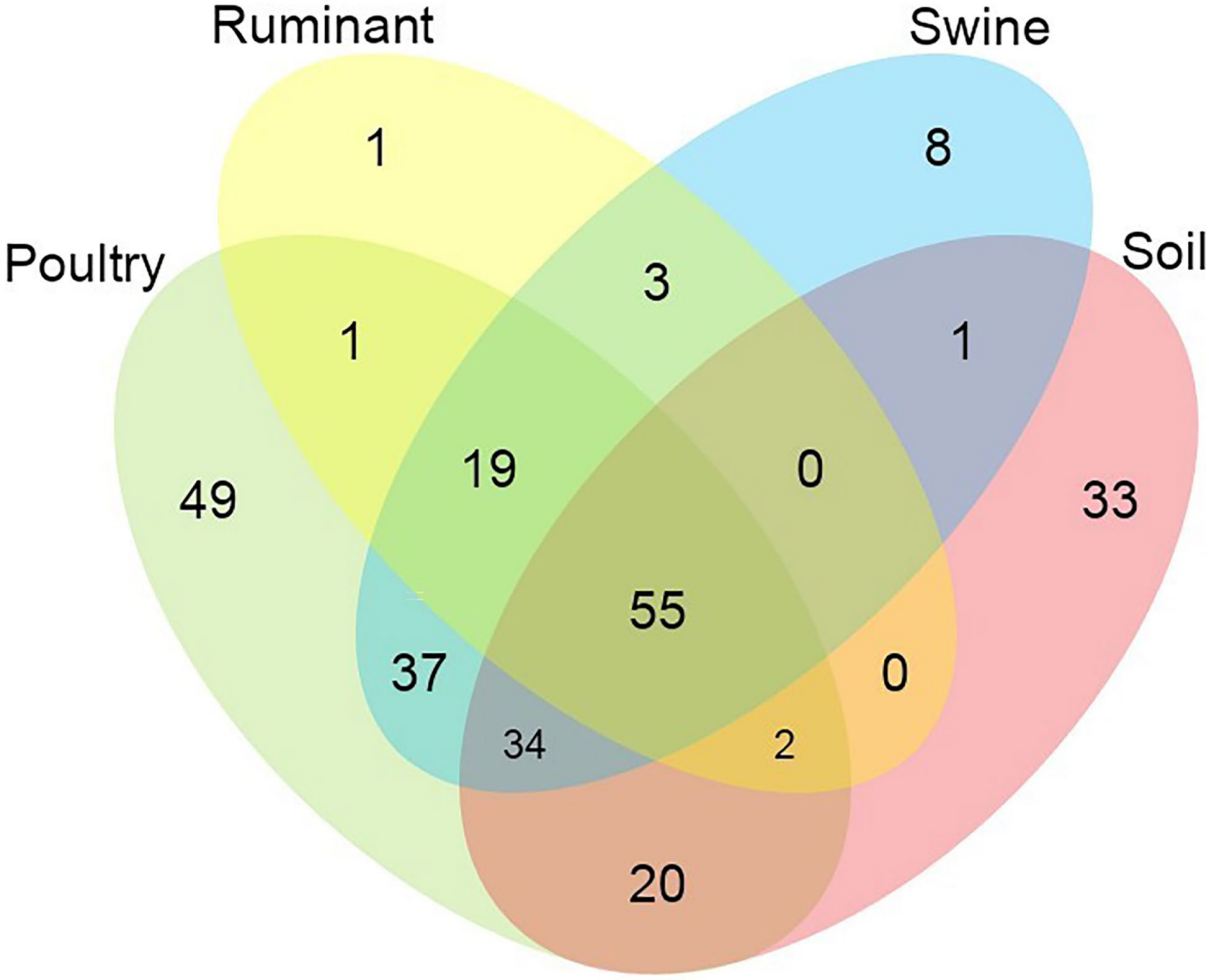
Venn diagram showing the number of shared and unique antimicrobial resistance genes among the analysed microbiomes.

**Figure 3:**
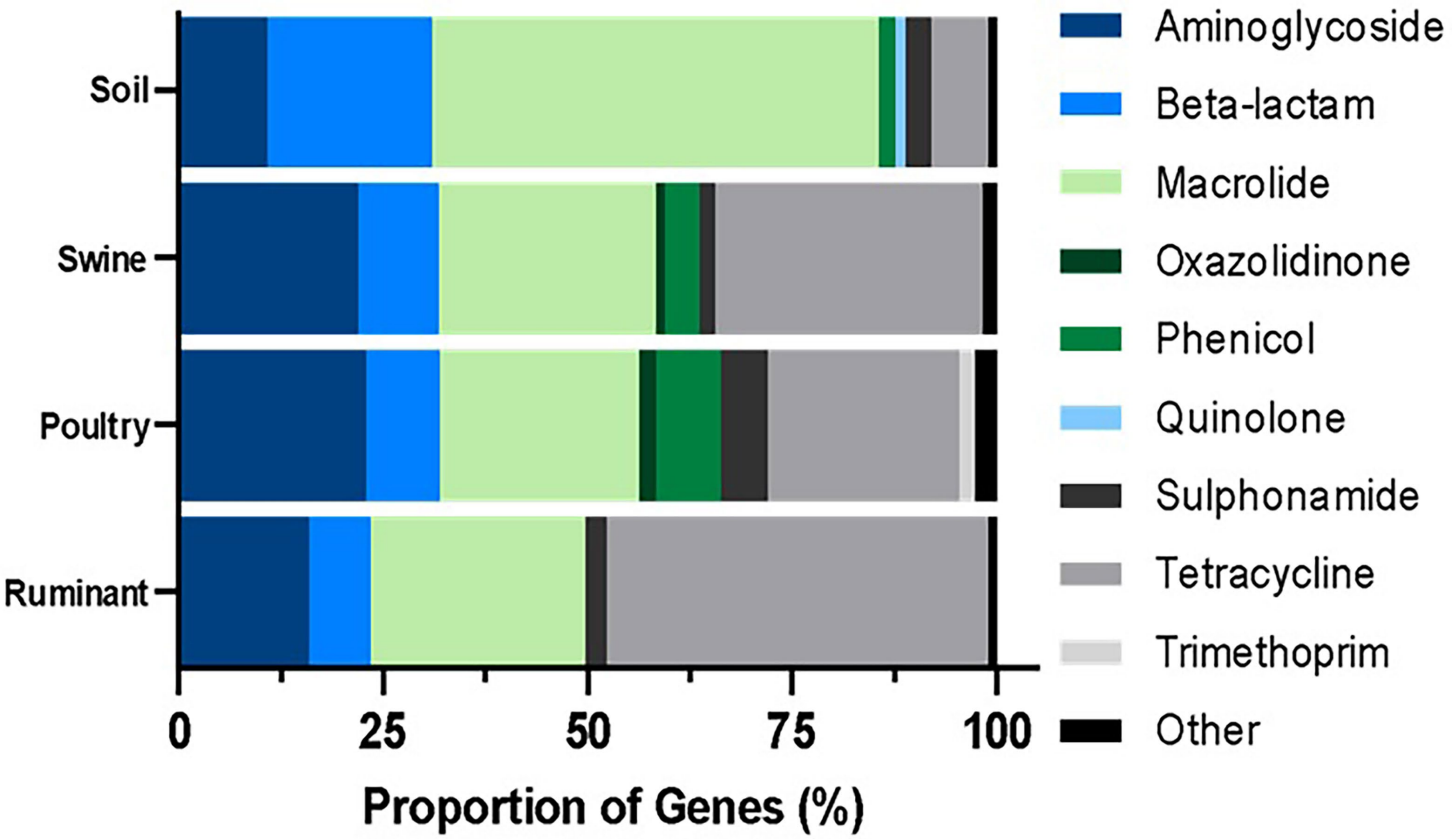
Percentage resistance genes grouped by antibiotic class per microbiome type. The ARG were identified by BLASTn and the proportion was estimated by the dividing the number of distinct resistance genes per antibiotic class by the total number of resistance genes for all classes.

**Figure 4:**
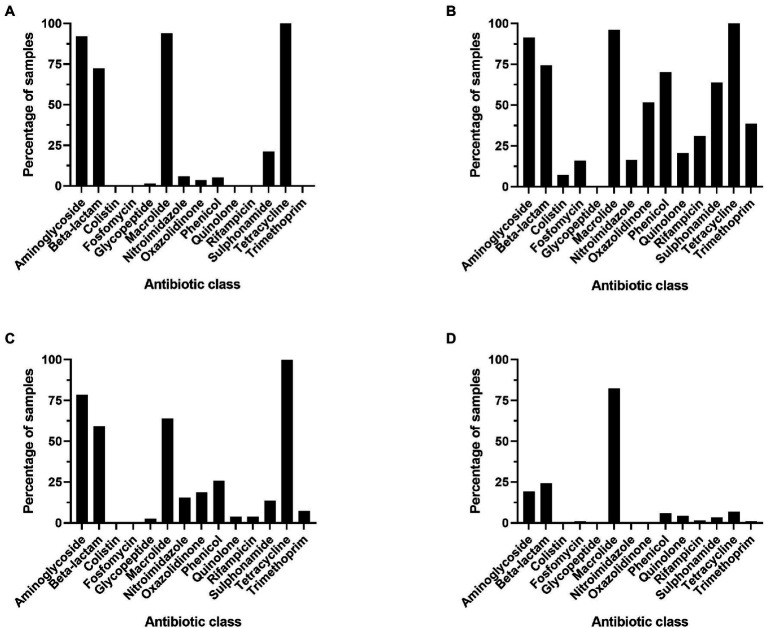
Percentage of metagenomes that contain at least one resistance gene per antibiotic class, genes were considered present if occurring >25 times in a sample. **(A)** Ruminant, **(B)** poultry, **(C)** swine, and **(D)** soil.

**Figure 5:**
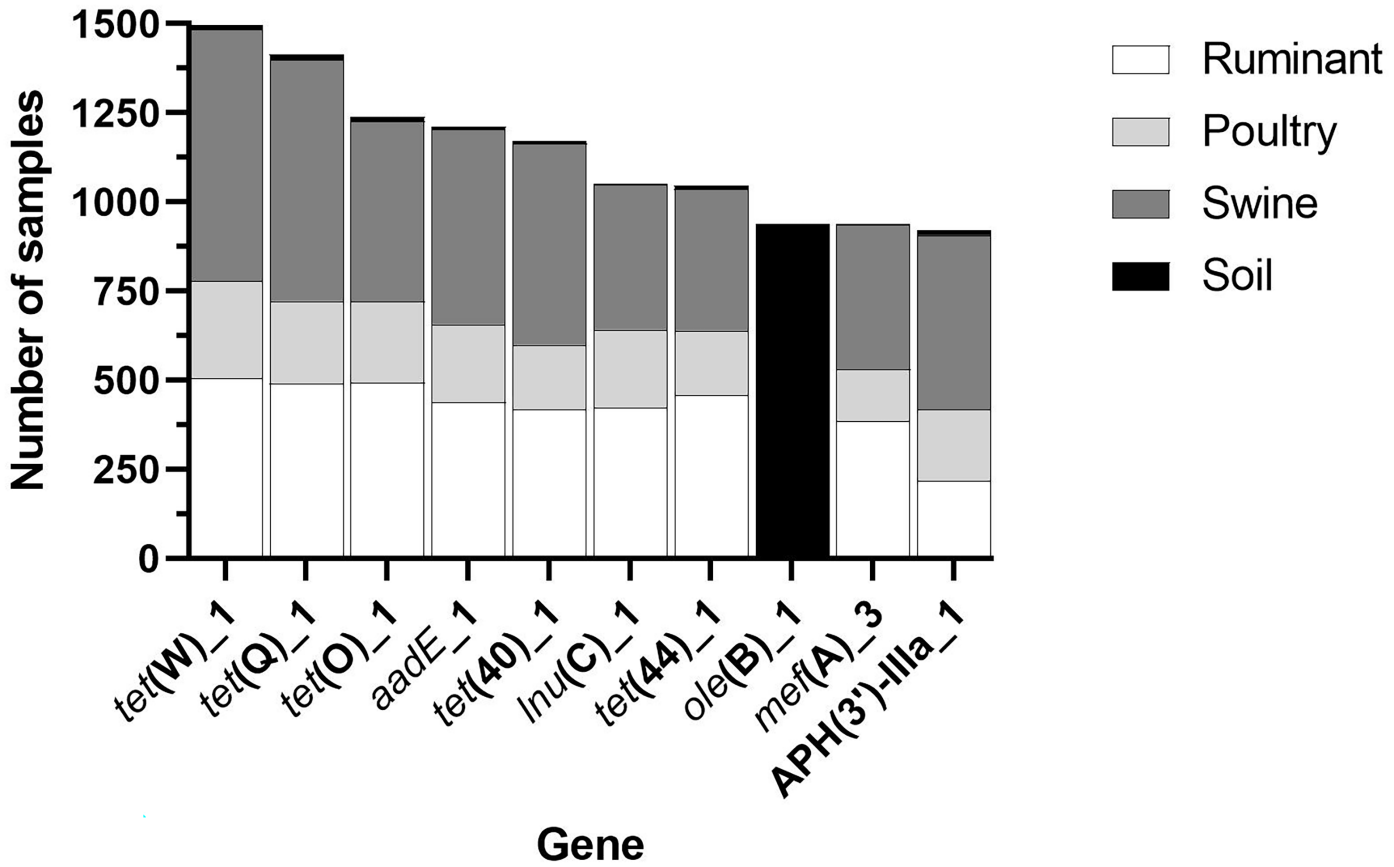
The most widespread resistance genes across all the four microbiome types.

**Figure 6:**
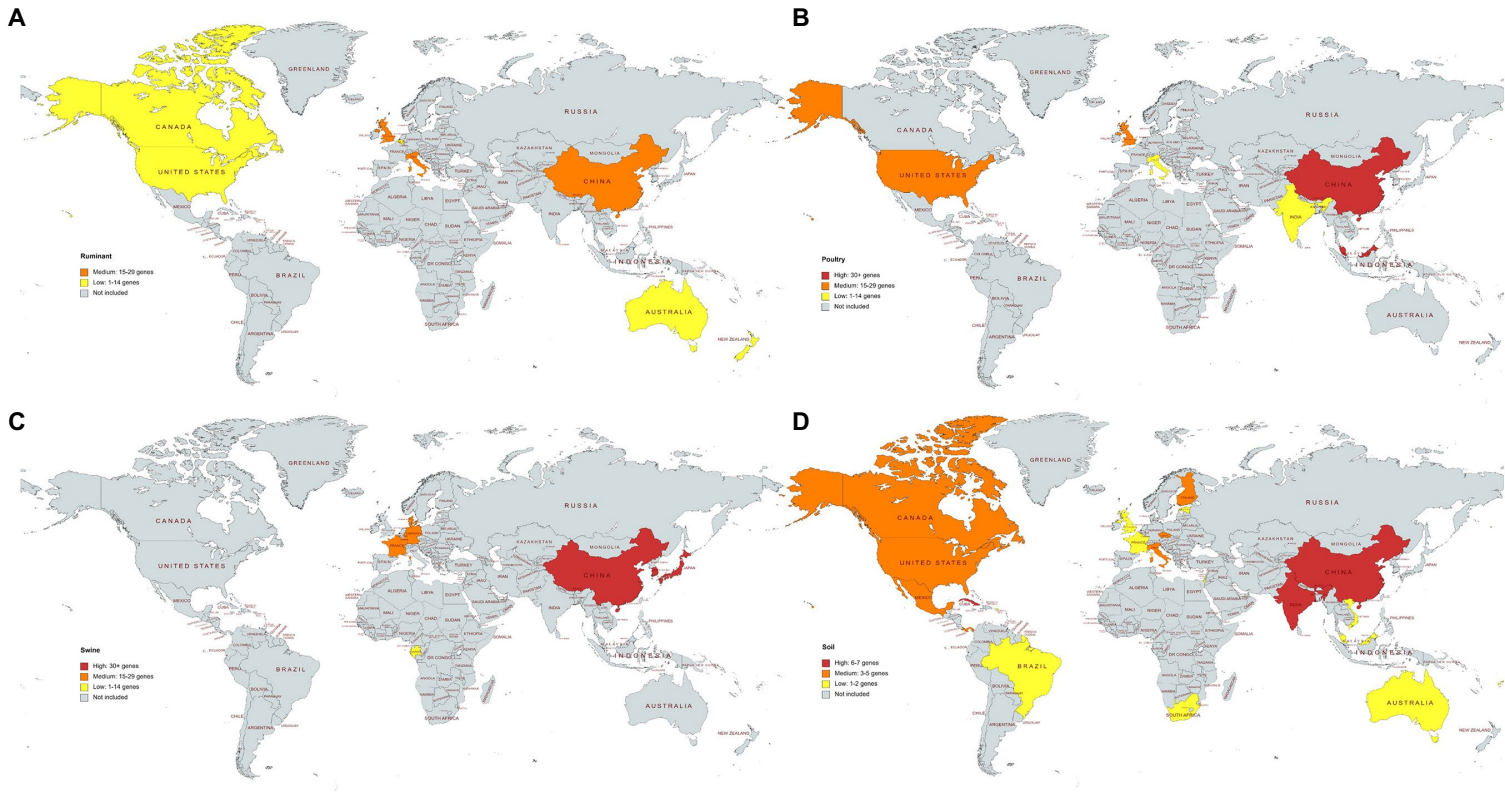
**(A–D)** The average number of different antimicrobial resistance genes per country and microbiome. Data for countries represented by one sample are not shown. **(A)** Ruminant, **(B)** poultry, **(C)** swine, **(D)** soil. **(A–C)** High: 30+ genes, medium: 15–29 genes, low: 1–14 genes. **(D)** Soil samples. High: 6–7 genes, medium: 3–5 genes, low: 1–2 genes. Soils samples from Antarctica are not included, and they showed medium average resistance (average of three distinct resistance genes per sample).

**Figure 7:**
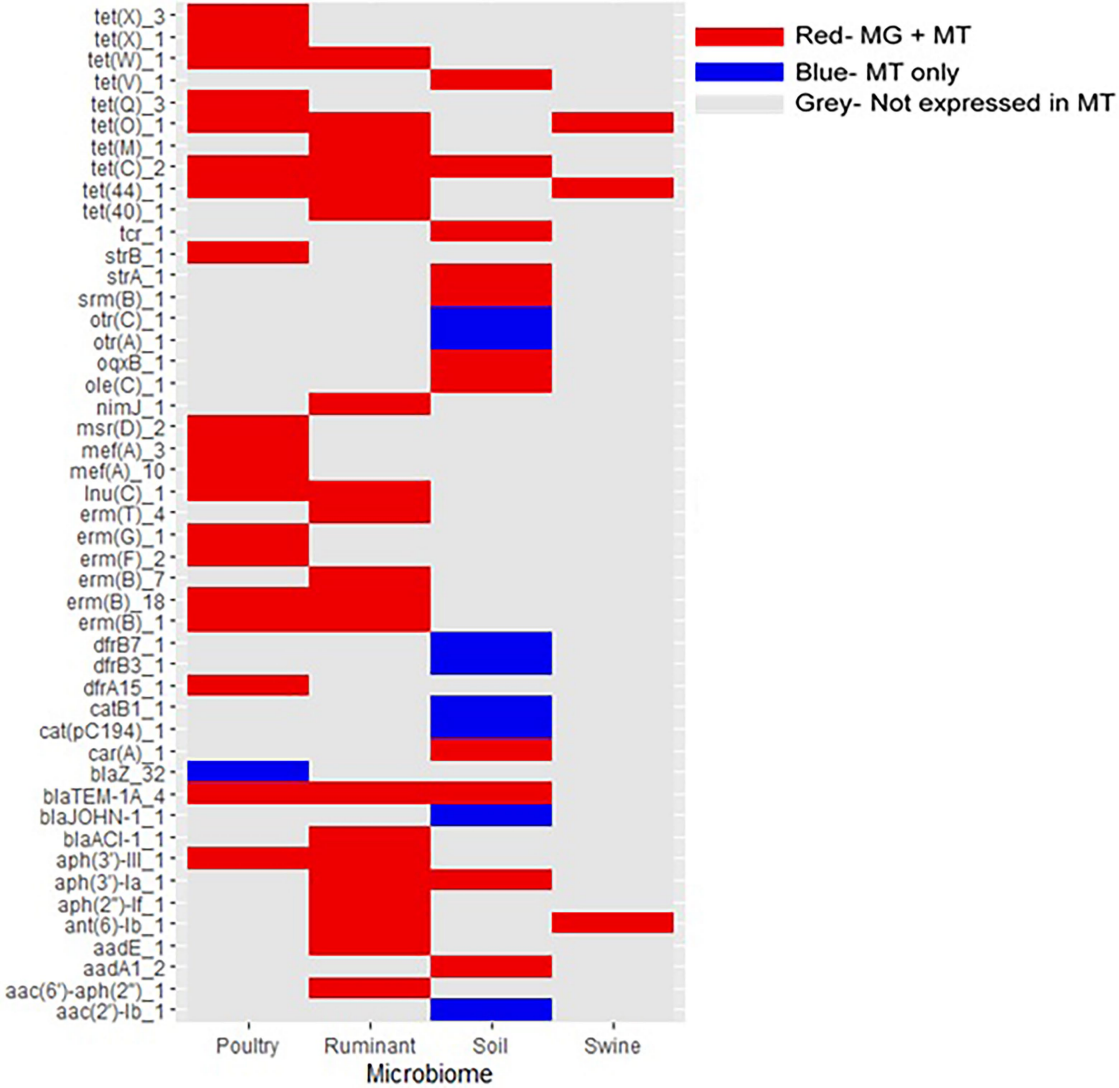
Expression of antimicrobial resistance genes in livestock and soil microbiomes. Only ARGs expressed in at least one metatranscriptome are included. MT, metatranscriptomes and MG, metagenomes. Red gene was present in both the metagenome and metatranscriptome datasets. Blue gene was present in the metatranscriptome dataset only. Gray gene was not present in metatranscriptomic datasets.

All statistical analyses were performed using GraphPad Prism version 9.1.0.221 (GraphPad Software, Inc., San Diego, CA), including Spearman r correlations, normality testing, chi-square, and ANOVA Kruskal-Wallis tests (significance at *p* < 0.05). Figure 1 was prepared using the My Maps function on Google maps (Google Maps, 2022). MyDraw version 5.0.0 software was used to produce Figure 2 (Nevron Software, 2020). Figures 3–5 were prepared using GraphPad Prism version 9.1.0.221 (GraphPad Software, Inc., GraphPad Software Inc., 10855 Sorrento Valley Rd.# 203., San Diego, CA 92121). Figure 6 was prepared using mapchart webpage (MapChart, 2022) and Figure 7 was prepared using RStudio (Wickham, 2016; RStudio Team, 2020).

## Results

### Summary of Resistance Within Soil and Livestock Microbiomes

Metagenomes from the soil environment and three livestock microbiomes [ruminant (species *Bos Taurus*), swine, and poultry] were selected from the NCBI SRA for resistome characterization. The soil microbiome was the largest dataset included, covering 28 countries, 4,161 samples, and 19,513 Gb of sequencing data. The livestock microbiomes covered 18 different countries, with ruminant samples from 8 countries, poultry samples from 6 countries, and swine samples from 7 countries. In this study, 1,682 livestock metagenomes and 7,659.5 Gb of metagenomic sequencing data were included (592 ruminant samples, 4,794.3 Gb, 306 poultry samples, 1,092.8 Gb, and 784 swine samples, 1,772.4 Gb). Acquired resistance genes contained within the ResFinder database were used to characterize the resistomes present within these four microbiomes.

A large range of ARGs were identified within each environment, the poultry microbiome datasets being the most diverse, with an average of 47 resistant genes identified per sample, significantly higher than the other microbiomes (*p* < 0.001). The soil dataset had the lowest average number of resistance genes per sample (4) and was significantly lower than all other microbiomes (adjusted *p* < 0.0001) (Table 3). Soil of agriculture origin and contaminated soil (antimony, arsenic, metal, mines, oil, pharmaceutical waste, polychlorinated biphenyl, and uranium) had significantly more different ARGs per sample than forest soil (p < 0.0001). On average forest soil samples contained three resistance genes per sample, and in comparison, agricultural soil had 8 ARGs per sample and contaminated soil had 5 ARGs. The highest number of resistance genes per sample, 73 ARGs, was observed in American soils that had been treated with manure. The ruminant microbiome contained the least number of distinct resistance genes (101), in comparison to the poultry, swine, and soil microbiomes that harbored 235, 167, and 182 different ARGs, respectively.

**Table 3:** Total antimicrobial resistance gene (ARGs) per microbiome.

	Number of samples	Resistance genes identified	Total occurrences of resistance genes	Avg. number of genes per sample (SEM)
Ruminant	509	101	8,248	16.20 (±0.365)
Poultry	277	235	13,210	47.69 (±1.875)
Swine	710	167	16,342	23.02 (±0.725)
Soil	1,548	182	5,930	3.83 (±0.146)

Summary showing the number of antimicrobial resistance genes (ARGs) identified per microbiome and the average number of ARGs identified in each group. Resistance genes identified refers to the number of distinctly different resistance genes identified across the microbiomes. Total occurrences of resistance genes was calculated as the number of incidences all resistance genes were identified in the microbiome. SEM, standard error of the mean.

In total, 55 AMR genes were shared between all environments, conferring resistance to six antibiotic classes (tetracycline, macrolide, aminoglycoside, beta-lactam, sulphonamide, and phenicols) (Figure 2). Almost one-third of these genes (18 of 55 genes) conferred resistance to tetracycline antibiotics, followed by macrolide resistance (13 of 55 genes) and aminoglycoside resistance genes (11 of 55). Poultry and soil microbiomes contained the largest number of distinct genes, which were not present in the other microbiomes (49 and 33 genes, respectively). The soil microbiome harbored 13 β-lactam resistance genes, which were not present in any other microbiome, while 11 aminoglycoside and 11 β-lactam resistance genes were present only in the poultry microbiome. The soil microbiome was also the only microbiome to contain the colistin-resistance genes, *mcr-7.1* and *mcr-5.1*. The ruminant and swine microbiomes contained the lowest numbers of distinct genes, ruminants contained one distinct resistance gene, *aph(2″)-If* conferring aminoglycoside resistance, while the swine microbiome contained 8 ARGs, which were not present in other microbiomes; aminoglycoside (*aph(2″)-Ih*, *rmtf*), beta-lactam (*bla*OXA-2, *bla*OXA-34, *bla*OXA-36) macrolide [*vga*(A)], and nitroimidazole (*nimB*, *nimH*). In total, 19 resistance genes were distinct to the livestock microbiomes and conferred resistance to the following antibiotic classes: β-lactam (5 genes), macrolide (5 genes), aminoglycoside (3 genes), phenicols (2 genes), tetracycline (2 genes), nitroimidazole (1 gene), and trimethoprim (1 gene). Poultry and swine microbiomes shared 37 resistance genes that were present in no other microbiome; this was the largest number of shared genes between microbiomes. These 37 genes conferred resistance to 11 different antibiotic classes (aminoglycoside, beta-lactam, phenicols, trimethoprim, quinolone, macrolide, colistin, nitroimidazole, oxazolidinone, rifampicin, and sulphonamide) and included the colistin resistance gene *mcr-1.11.*

### ARG Abundance and Diversity in Microbiomes by Antibiotic Class

The majority (54.70%) of genes identified in the soil environment encoded resistance against macrolide antibiotics (Figure 3). The most widespread macrolide-resistant gene within this environment was *ole*(B)_1, which was identified in 930 soil samples (60.08% of analyzed soil samples). This gene was spread across 73 different soil types including agriculture, desert, forest, grasslands, garden, permafrost, and soils, both with and without crop. Macrolide resistance genes were also the most prevalent in poultry samples with over 24.38% of ARGs identified in this microbiome conferring resistance against macrolides. The most widespread gene within poultry metagenomes was the *erm*(B)_7 gene, identified in 256 samples (92.41%). In contrast, the most commonly identified class in both swine and ruminant environments were tetracycline resistance genes, which represented 46.64% and 32.71% of resistance genes identified in ruminant and swine microbiomes, respectively (Figure 3). The most commonly identified tetracycline resistance gene in both environments was *tet*(W)_1, identified in 99.41% of ruminant samples and 99.44% of swine samples.

The resistome profiles identified in each of the four microbiomes were significantly different (*p* < 0.0001). The macrolide resistance genes were widespread across all microbiomes, identified in >80% of all samples. Tetracycline and aminoglycoside resistance genes were widespread across all livestock microbiomes but were present in small proportion of soil samples (6.85% of samples contained at least one tetracycline resistance gene). Also prevalent in livestock samples were β-lactam antibiotic resistance genes, which were identified in more than half of livestock microbiome samples. Resistance genes against colistin, fosfomycin, oxazolidinone, phenicol, quinolone, rifampicin, sulphonamide, and trimethoprim were present in more poultry samples than in any other microbiome (Figure 4).

Three antibiotic classes were represented in the 10 most common genes, including tetracycline [*tet*(W)_1, *tet*(Q)_1, *tet*(O)_1, *tet*(40)_1 and *tet*(44)_1], macrolide (lincosamide-streptogramin B) [*lnu*(C)_1, *ole*(B)_1, *mef*(A)_3], and aminoglycoside [*aadE*_1, APH(3′)-IIIA_1] (Figure 5). Fifty percent of the most widespread genes conferred tetracycline resistance. All genes, except for *ole*(B)_1, were identified in a large number of livestock microbiome samples but were not widespread in soil samples (Figure 5). The macrolide resistance gene, *ole*(B)_1, was identified in 939 (60.66%) of soil samples but was only identified in 3 poultry and 6 ruminant samples and was not identified in any swine samples. Also, widespread across soil samples was the macrolide resistance gene, *tlr(C)_1*, identified in 589 soil samples.

### ARG Abundance in Microbiomes by Country of Origin

Metagenomes for all four microbiomes sourced from China were analyzed. Chinese soil, poultry and swine microbiomes were classified as containing high number of average resistance genes per sample, while the Chinese ruminant microbiomes showed medium resistance levels. The United States showed medium resistance levels in both the soil and poultry microbiomes and low resistance in the ruminant microbiome (Figure 6). All European swine microbiomes showed medium resistance. The ruminant and poultry microbiomes selected from the United Kingdom also showed medium levels of resistance genes. Cuba, India, and China soil microbiomes showed high resistance in comparison to other countries, but only one metagenome sequence was available for Cuba (Figure 6). In general, soil samples across all the countries harbored less AMR genes than the livestock microbiomes.

### Expression of ARGs in Microbiomes

Metatranscriptomic data for each microbiome was used to ascertain whether the ARGs were expressed and thus functionally active (Figure 7). The expression of ARGs, previously identified in the metagenome datasets, were confirmed in metatransciptomes for all four of the microbiomes. Of the 76 poultry metatranscriptomic sequences, 72 contained ARGs (94.7% of sequences). Of the 32 ruminant metatranscriptomes, 19 contained ARGs (59.4%), similarly 46.8% of soil metatranscriptomes contained ARGs (22 of 47 metatranscriptomes), while ARGs were identified in only 3 of the 60 swine metatranscriptomes analysed (5.0%).

All of the ARGs expressed in the ruminant and swine metatranscriptomes were identified in the associated metagenomes, similarly 19 of the 20 ARGs identified in the poultry metatranscriptomes were present in the poultry metagenomic sequences. Of the 18 ARGs expressed in the soil metatranscriptomes, 11 were also identified in the soil metagenomic dataset. The percentages of ARGs identified in metagenomes that were also identified in metatranscriptomic data are as follows: 8.1% poultry, 19.8% ruminant, 1.8% swine, and 6.0% soil. The expression of ARGs in the microbiomes did report similarities, with aminoglycoside and tetracycline genes being expressed in all of the microbiomes. Additionally, ruminant, poultry, and soil microbiomes all expressed β-lactam, macrolide, and quinolone ARGs.

## Discussion

In this study, we identified the acquired ARGs in the soil environment and the gastrointestinal tracts of livestock animals including ruminants, swine, and poultry. We aimed to take a global approach to assess the resistomes in these four microbiomes by using publicly available metagenomic datasets on the NCBI SRA database and metatranscriptomic sequencing data from 37 different countries. The four microbiomes investigated in this study contained a large diversity of ARGs, with each environment containing over 100 different resistance genes, conferring resistance to at least 11 different antibiotic classes.

The livestock microbiomes, namely, poultry, ruminant, and swine, showed similarities in their resistomes, with ARGs against tetracyclines, aminoglycosides, and β-lactam antibiotics being widespread among livestock microbiomes but less abundant in the soil microbiomes. The soil microbiome also showed very low average number of ARGs per sample (4 genes) in comparison to the livestock microbiomes (25 genes). This study provides a global and more expansive insight and is in agreement with previous findings that soil in general had lower ARG abundance than livestock microbial communities (Pal et al., 2016).

Tetracycline genes were spread across all livestock microbiomes, particularly, the tetracycline resistance genes *tet*(Q)_1 and *tet*(W)_1. Both of these tetracycline genes have been identified in important human pathogens including *Prevotella* spp. and *Clostridium difficile* (Arzese et al., 2000; Spigaglia et al., 2008). The *tet*(W)_1 gene, identified in the rumen anaerobe *Butyrivibrio fibrisolvens* has also been identified in human feces (Scott et al., 2000). The potential widespread distribution of this gene could be due to it’s ability to use horizontal gene transfer through association with integrative and conjugative elements (ICE) including the newly identified ICE_RbtetW_07 (Sabino et al., 2019). Tetracycline genes were also confirmed to be expressed in each of the four microbiomes, with *tet*(44) and *tet*(O)_1 expressed in all livestock microbiomes. The extensive use of tetracycline antibiotics, extensive host range seen for the *tet* resistance gene and the genes associations with mobile genetic elements such as ICEs may all contribute to the widespread identification of these genes in this study (Spigaglia et al., 2008).

The poultry microbiome was the most diverse, harboring the highest number of different ARGs and contained the highest number of unique ARGs. So far, research has been relatively limited on the poultry resistome and how it compares to other livestock microbiomes (Ma et al., 2021), therefore this study sheds new light on the poultry resistome. Such high resistance in poultry farming could be due to the high antibiotic usage associated with the widespread intensive farming used in this livestock sector (Kirchhelle, 2018).

Additionally, to the livestock microbiomes, agriculture soil also showed high numbers of ARGs. Specifically agricultural soils that had been fertilized using manure showed particularly high numbers of ARGs. The spread of ARGs in soil due to manure application has been considered a serious public concern (Checcucci et al., 2020). The most widespread resistance gene within the soil samples was *ole*(B)_1, this gene was not widespread across the livestock microbiomes. The *ole*(B)_1 gene confers resistance against oleandomycin through the production of a ribosome protection protein (Kerr et al., 2005). Oleandomycin is an active ingredient of a last resort drug used to treat mastitis in cattle where other treatments have failed (Zoetis, 2016). This study highlights that the *ole*(B)_1 resistance gene is widespread in different soil types including agriculture, desert, forest, grasslands, garden, permafrost, and soils both with and without crop, and builds upon previous identifications of this resistance gene in arable farmland soil, forest soil, and compost from Poland (Popowska et al., 2012). The *ole*(B)_1 gene has been identified in zoonotic pathogens within soil and is also widely distributed in the human gut (Yang et al., 2016). The gene product of *ole*(B)_1 is a member of the ABC-transporter superfamily, and in addition to conferring antibiotic resistance, members of this gene family are also involved in transport, DNA repair, enzyme regulation, and translational control (Davidson et al., 2008; Sharkey et al., 2016). Therefore, although the *ole*(B)_1 genes found in the soil samples in this study may confer resistance in these environments, their gene products may also serve other functions in that environment, highlighting that ARG hits within samples does not always equal resistance.

The majority of the metagenomic studies included in this analysis did not have associated metatranscriptomic data. Therefore, we employed the most suitable alternative methods and analyzed the largest publicly available metatranscriptomic dataset for each microbiome to explore if the ARGs identified within the metagenomes could be expressed in their associated microbiomes. Low numbers of the ARGs identified in the metagenomes were identified in metatranscriptomes, we suggest this is due to the much smaller amount of metatranscriptomes analyzed in comparison to the large number of metagenomes. Resistance genes expressed in the four microbiomes were diverse covering eight different antibiotics classes. In total, 28 ARGs were expressed in the livestock microbiomes that were not expressed in the soil microbiome, suggesting these genes are more adapted to expression in the gut microbiome.

Resistance to one of the last resort drugs, colistin, was detected in poultry, swine, and soil microbiomes. In total, 20 poultry metagenomes (7.22% of poultry samples) contained colistin resistance genes, with the *mcr-1.11* gene being the most widespread. Of the 20 colistin ARG containing poultry samples, 19 contained the *mcr-1.11* gene and one sample contained the *mcr-3.1* gene. The *mcr-1.11* was also the only colistin-resistant gene identified in swine microbiome. The *mcr-1.11* gene is a variant of the *mcr-1* gene, which likely emerged due to spontaneous mutation within a plasmid structure (Deshpande et al., 2019). The plasmid-borne *mcr-1* gene and its variants pose a significant challenge to human healthcare, as colistin and the polymyxins are currently last resort treatments against serious multidrug resistant Gram-negative human pathogens (Gao et al., 2016). In this study, livestock microbiome datasets from China and soil microbiome datasets from the United States harbored the *mcr-1.11* gene and the *mcr-5.1 and mcr-7.1* colistin-resistant genes, respectively. These soils included both agriculture and uncultivated soils. Both the *mcr-5.1 and mcr-7.1* genes are associated with mobile genetic elements including transposons and plasmids and both were identified in human pathogens (Han et al., 2006; Borowiak et al., 2017). The *mcr-5.1* gene has been identified in *Salmonella. enterica* subsp. *enterica* serovar Paratyphi B dT+ (*S. Paratyphi* B dT+; formerly called *Salmonella Java*), which causes gastroenteritis and is currently an emerging problem worldwide (Han et al., 2006; Borowiak et al., 2017). While the *mcr-7.1* gene has been identified from serious human pathogen *Klebsiella pneumoniae* (Yang et al., 2018). Previous studies have identified the *mcr-5.1* and *mcr-7.1* genes in Chinese soil (Zheng et al., 2017; Shen et al., 2018; Anyanwu et al., 2020). However, this study also shows for the first time that they are present in soil from the USA.

Vancomycin-resistant genes were identified across all four microbiomes; vancomycin is an important treatment option in endocarditis and other serious infections including the treatment of methicillin-resistant *Staphylococci* infections (Bennett et al., 2019). The swine microbiome harbored the largest diversity, containing four different vancomycin resistance genes: *vanG*, *vanA-B*, *vanH-B*, and *vanXY-G*. Vancomycin resistance was more widely disseminated than colistin resistance, with vancomycin resistance genes identified in 10 counties including Canada, China, Denmark, England, France, Germany, Japan, New Zealand, South Korea, and United States.

A large number of metagenomic sequences were available from China, and it was the only country to have samples included in each of the four microbiomes. In this study, medium levels of ARGs were observed within the ruminant microbiome and high resistance levels in the poultry, swine, and soil microbiomes. High ARGs occurrence in different livestock environments have been previously reported in China (Wang et al., 2016; Qiao et al., 2018). The Chinese Ministry of Agriculture have aimed to reduce the use of veterinary antibiotics through their “National Action Plan for Restraining Bacteria of Animal Origin” (Ministry of Agriculture of the People’s Republic of China, 2017). One objective of the action plan is to improve the monitoring system of veterinary antibiotics including the implementation of regulatory actions to strengthen the supervision and management of veterinary antimicrobials (Ministry of Agriculture of the People’s Republic of China, 2017).

The microbiomes included from the United Kingdom and United States all showed low to medium average number of resistance genes per sample. The poultry and ruminant microbiomes from the United Kingdom showed medium resistance levels, as did poultry and soil microbiomes from the United States. Both the United Kingdom and United States have launched AMR action plans aimed at reducing antimicrobial consumption and AMR (HM Government, 2019; Federal Task Force on Combating Antibiotic-Resistant Bacteria, 2020). The United Kingdom has seen large reductions in antibiotic usage in animals, with a 52% reduction of antibiotics usage for food-producing animals observed from 2014 to 2019 (UK-VARSS, 2020). Although low to medium levels of ARGs in livestock microbiomes were observed in United States, the United States does not have such reductions in antibiotic consumptions as those reported in the United Kingdom. However, the United States are introducing guidance in the prescription of antibiotics in the livestock sector including the implementation of the FDA’s Guidance for Industry #213 (CDC, 2021). Differences in countries ARG levels could be due to varying regulations on antibiotic use.

All four microbiomes shared 55 AMR genes in common, with a number of genes being widespread across the sample types, stressing the need for a One Health approach to AMR surveillance encompassing both livestock and environmental microbiomes in addition to human AMR surveillance. This study successfully used available metagenomic and metatranscriptomic data to identify the ARG reservoirs present in four microbiomes across 37 countries (no ARGs were present in the analysed datasets from five of these countries). AMR and ARGs have been identified as a critical human health challenge (WHO, 2019). By expanding our knowledge on the resistomes present in different systems such as those included in this study, policies supporting issues such as antimicrobial usage and applications to soil, e.g., manure can be better informed. As highlighted in this study, ARGs are both persisting and being expressed in the microbiomes with microbiomes from many countries harboring high number of ARGs, and this is a global problem. Therefore, in addition to a One Health approach, a global approach must be employed to aid our understanding of ARG abundance and diversity globally, and so improve our ability to tackle the AMR problem in a holistic manner.

## Data Availability Statement

All metagenomic sequences are freely available on the NCBI SRA, while metatranscriptomics were sourced from the FAANG database and ENA database. Raw data is provided in the Supplementary Table 1. The original contributions presented in the study are included in the article/Supplementary Material; further inquiries can be directed to the corresponding author.

## Author Contributions

KL performed the analysis with guidance from CC, FS, and SH. FR assisted with metatranscriptomic work. FS and LO contributed significantly to manuscript preparation. SH is PI of KL and oversaw each stage of this study. JM and SM are also supervisors to KL. All authors contributed to the article and approved the submitted version.

## Funding

This work was supported by the Northern Irish Department of Agriculture, Environment and Rural Affairs.

## Conflict of Interest

The authors declare that the research was conducted in the absence of any commercial or financial relationships that could be construed as a potential conflict of interest.

## Publisher’s Note

All claims expressed in this article are solely those of the authors and do not necessarily represent those of their affiliated organizations, or those of the publisher, the editors and the reviewers. Any product that may be evaluated in this article, or claim that may be made by its manufacturer, is not guaranteed or endorsed by the publisher.

## Supplementary Material

The Supplementary Material for this article can be found online at: https://www.frontiersin.org/articles/10.3389/fmicb.2022.897905/full#supplementary-material

## Footnotes

^1^ https://data.faang.org/home

^2^ https://www.ebi.ac.uk/ena/browser/
