# Supplementary figures and images for: Resistome Analysis of Global Livestock and Soil Microbiomes
Source: Front Microbiol. 2022 Jul 7;13:897905. doi: 10.3389/fmicb.2022.897905 (PMC9300982; doi:10.3389/fmicb.2022.897905)

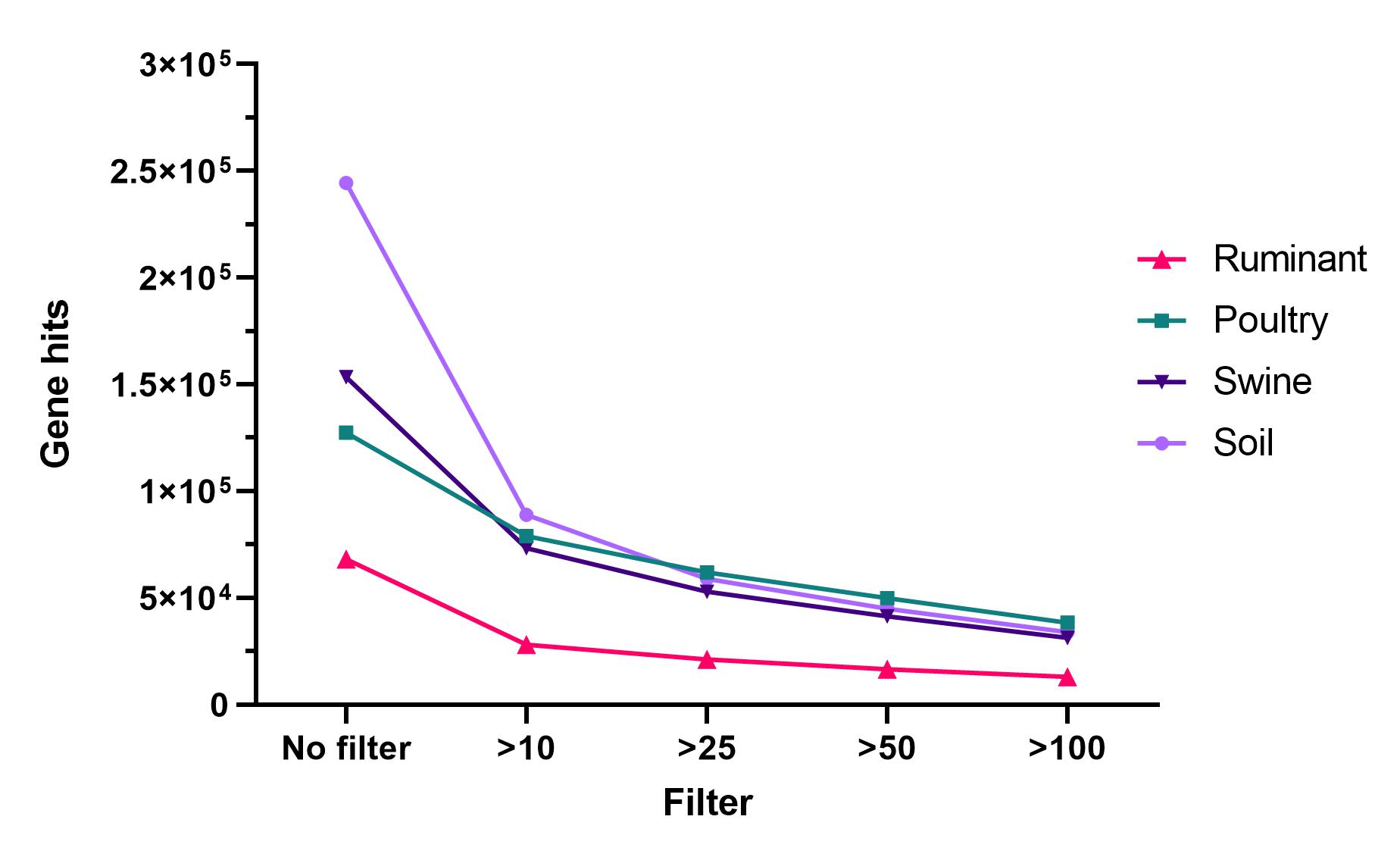

Supplement: Supplementary file 2 [file Image_1.JPEG]
